# Tectonic structures of the Dome Fuji region, East Antarctica, based on new magnetic data

**DOI:** 10.1038/s41598-024-69471-8

**Published:** 2024-08-10

**Authors:** Alexandra Guy, Graeme Eagles, Olaf Eisen

**Affiliations:** 1 Czech Geological Survey, Prague, Czech Republic; 2 Alfred-Wegener-Institut Helmholtz-Zentrum Für Polar Und Meeresforschung, Bremerhaven, Germany; 3 Fachbereich Geowissenschaften, Universität Bremen, Bremen, Germany

**Keywords:** Magnetic data and analysis, Subglacial topography, Gravity analysis, Tectonic, Antarctica, Gondwana, Geophysics, Tectonics

## Abstract

The Oldest Ice Reconnaissance (OIR) airborne geophysical survey in East Antarctica was flown over approximately 170,000 km^2^ of the Dome Fuji region in 2016/17. The survey’s results support new insights into the subglacial geology and its meaning for the tectonic histories of the supercontinents Rodinia and Gondwana. The new magnetic and radar-derived bed topography data are integrated with previously acquired magnetic and gravity data, allowing the mapping of crustal domains within and beyond the survey’s limits. The magnetic data reveal three distinct domains within the survey region, delineated by N–S oriented boundaries, partly aligned with gravity domains following upward continuation transformations for both datasets. Additionally, four primary sets of magnetic lineaments were identified, exhibiting correlations with topographic and gravity patterns. These correlations indicate the continuation of the Tonian Oceanic Arc Super Terrane (TOAST) southward of its previously known southern limit. Moreover, an E–W-trending magnetic anomaly, the Elbert magnetic anomaly, suggests the suture between the recently-proposed subglacial Valkyrie craton and the TOAST. Furthermore, the analysis reveals a broad scale shear zone, named here the OIR shear zone, which formed as a result of oblique collision of the Ruker and Valkyrie cratons during the amalgamation of Gondwana.

## Introduction

**Figure 1:**
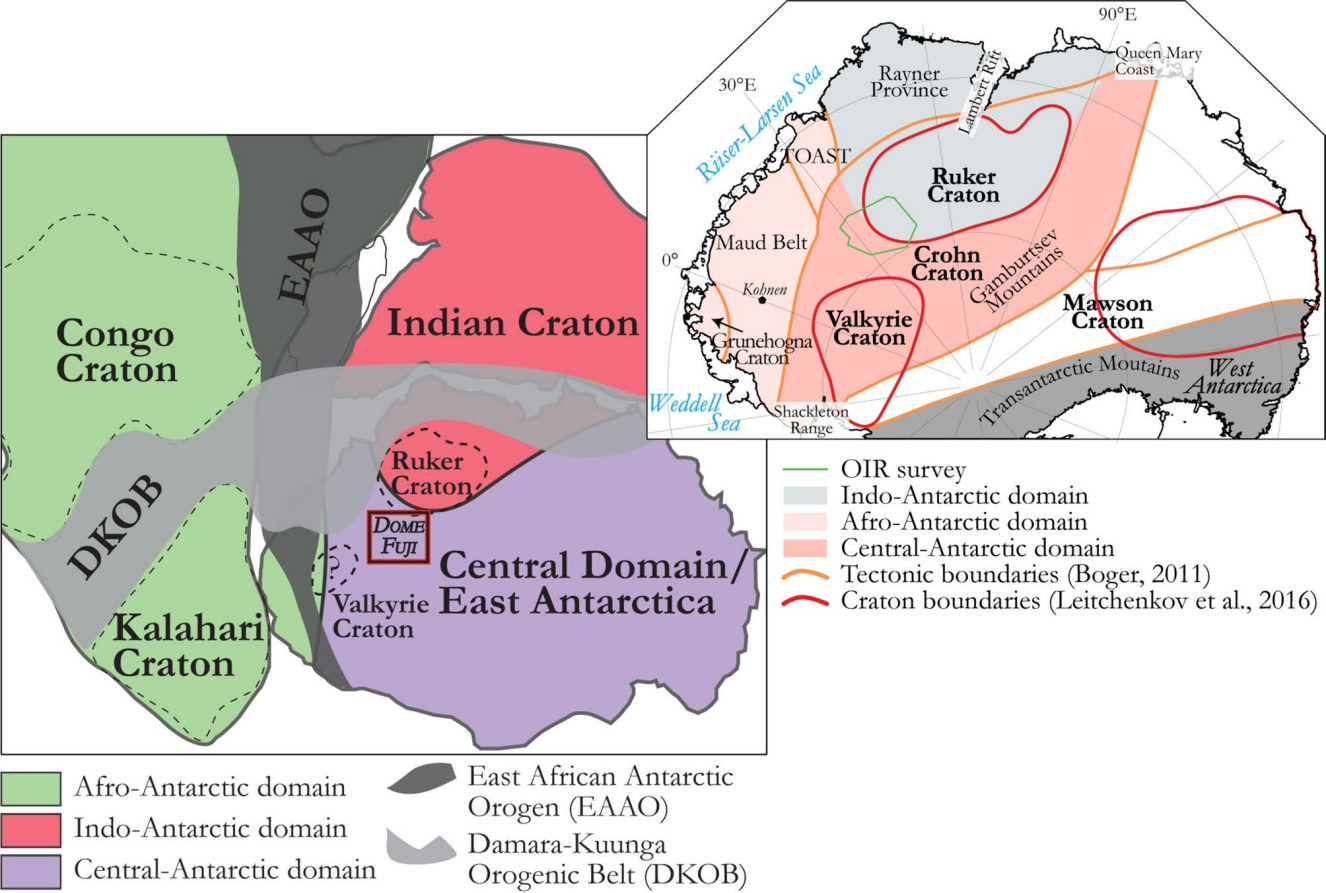
Location of the OIR magnetic data at a crossroads of three tectonic domains of East Antarctica and within the East African-Antarctic Orogen. Inset, right: summaries of two recent contrasting attempts to map large scale geological structures in the Antarctic interior. New artwork created for this manuscript based on published interpretations^2,3,5,25^ and using Adobe Illustrator 2023 (https://www.adobe.com/products/illustrator.html).

Parts of East Antarctica retain a record of the Mesoproterozoic-Neoproterozoic (1.3–1.0 Ga) amalgamation of cratons to form the supercontinent Rodinia^1^, and its later breakup. Many of the fragments of Rodinia were subsequently reworked by Neoproterozoic-Cambrian (~ 650–490 Ma) orogenesis during the amalgamation of Gondwana^2,3^, and afterwards experienced the supercontinent’s Jurassic–Cretaceous (180–130 Ma) breakup^4^. Detailed interpretations of these events rely on studies of scattered outcrops at nunataks located within a few hundred kilometres of the coast, and on correlations with better exposed cratonic and peri-cratonic rocks in East Antarctica’s neighbours in Gondwana, Africa, India, Australia, and South America (Fig. 1). Further inland, where rocks with similar affinities are likely to be juxtaposed with each other and possibly with other ‘central Antarctic’ cratonic regions, there is no outcrop and boreholes have yet to sample any rocks from beneath the ice sheet. Interpretations of the East Antarctic interior, consequently, differ enormously (inset, Fig. 1)^3,5^. Indirect geophysical methods help to characterize this region for a better understanding of the basement architecture, and hence of tectonic processes responsible for Gondwana’s amalgamation and Rodinia’s amalgamation and breakup^6–11^.

**Figure 2:**
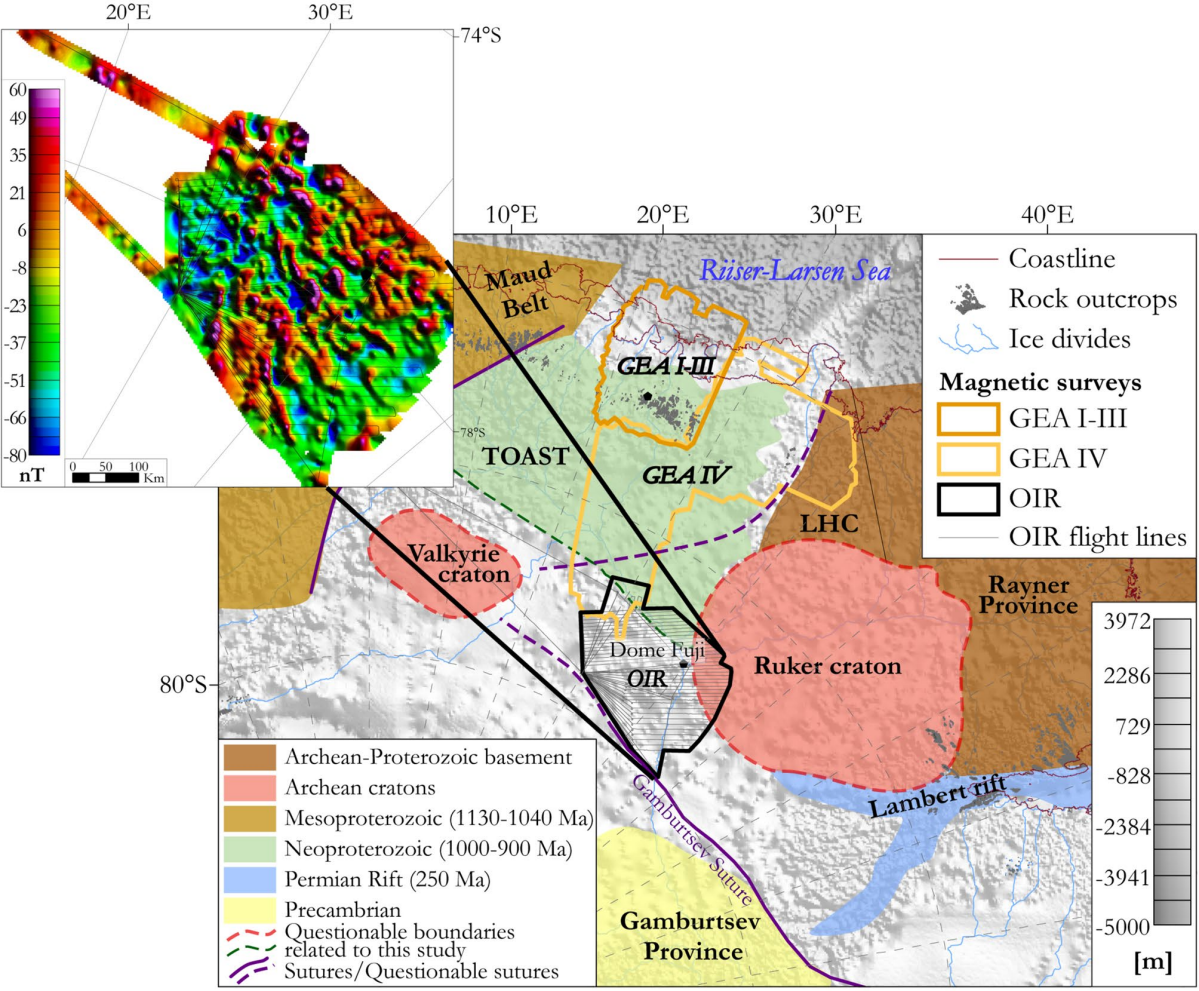
Tectonic map based on previous geological interpretations in Dronning Maud Land (modified from refs^9,11^) superimposed on subglacial topography^33^. Inset: preview of the OIR total field magnetic data. TOAST-Tonian Oceanic Arc Super Terrane; GEA-Geodynamic Evolution of East Antarctica; LHC: Lützow-Holm Complex. Proposed sutures, including the Gamburtsev^34^ and LHC suture^3,32^, are also shown. The inset map was built using Geosoft Oasis Montaj 9.6 (https://www.seequent.com/products-solutions/geosoft-oasis-montaj/), the tectonic map was generated using ArcMap 10.6.1 (https://www.esri.com/en-us/arcgis/products/arcgis-desktop/resources) and Adobe Illustrator 2023.

Known from nunataks and mountain ranges, the area of African affinities north and west of Dome Fuji includes the Grunehogna Craton and the Maud Belt, its 1130–1040 Ma collisional boundary with Antarctic provinces further east and south^3,18–23^ (Fig. 2). The Maud Belt was largely reworked at ~ 650–490 Ma as the western front of the late Neoproterozoic–early Cambrian East African-Antarctic Orogen (EAAO; Fig. 1), a prominent member of the Pan-African family of orogenies that effected Gondwana’s amalgamation^27^. East of the Maud Belt, an ice-covered area of subdued magnetic response in sparse regional airborne profiles has been interpreted as a further craton with unknown affinities and age, the Valkyrie Craton^15,18^. East of Dome Fuji, a domain of Indo-Antarctic affinities^2,3^ is occupied by a subglacial craton or cratons referred to as the East Antarctic^12–14^, Crohn^3^, or Ruker^15^ craton(s), which are defined from exposures of Paleoarchean to Proterozoic rocks in the southernmost Lambert Rift^23,28^. The Rayner Province is exposed near the coast to the north of this domain, and represents its collision with Indian cratons in the 1400–900 Ma period^16,17^. Along the coast to the west of the Rayner Province, the exposed Lützow-Holm Complex is composed of Archean-Proterozoic basement amalgamated from magmatic arcs and oceanic material during late Neoproterozoic to early Cambrian Pan-African orogeny^29–31^ that post-dates the EAAO, and whose supposed suture continues from the western margin of the complex into the ice-covered Central Antarctic domain^3,32^ (Figs. 1, 2). North of Dome Fuji, sandwiched between the Afro-Antarctic and Indo-Antarctic domains^21^ (Fig. 2), lies the Tonian Oceanic Arc Super Terrane (TOAST; 1000–900 Ma^24–26^), a broad zone of early Neoproterozoic juvenile arc-related crust^24,25^ interpreted to originate from the Proterozoic Mozambique Ocean^10,21,30^, which separated the eastern and western parts of what was to become Gondwana. TOAST rocks associate with a distinctive pattern of medium-amplitude NW–SE-trending magnetic lineaments that continues for hundreds of kilometres inland of where they crop out^9,11,24–26^. Southeast of Dome Fuji, the subglacial Gamburtsev Mountains represent a third domain of unknown or Central Antarctic affinities. The Central Antarctic domain has been interpolated over long distances between outcrops along the Queen Mary Coast, Shackleton Range, and at the southern end of the Lambert Rift^3^. Dome Fuji therefore lies at the crossroads of the Afro-Antarctic, Indo-Antarctic and Central-Antarctic domains of cratonic associations, in a pivotal position for understanding processes of supercontinent formation and breakup in Precambrian times.

This study presents a new 3 km spaced grid of aeromagnetic data in the Dome Fuji region, which was previously so sparsely surveyed that a detailed interpretation of its subglacial geology was impossible. It analyses the new data together with previously-acquired magnetic, subglacial topography, and gravity anomaly data from the survey region and its surroundings. The analysis aims at defining crustal domains and their boundaries from geophysical signals, leading to a comprehensive geophysical and tectonic description of the region’s crustal architecture.

## New interpretations of potential field data from the Oldest Ice Reconnaissance survey area

To discriminate, complement, and modify the existing interpretations of East Antarctic basement geology described in previous studies, we concentrate on new and existing airborne geophysical data from the region around Dome Fuji. The new data were collected as part of the OIR survey in 2016–2017, which is described in detail in the Methods section.

**Figure 3:**
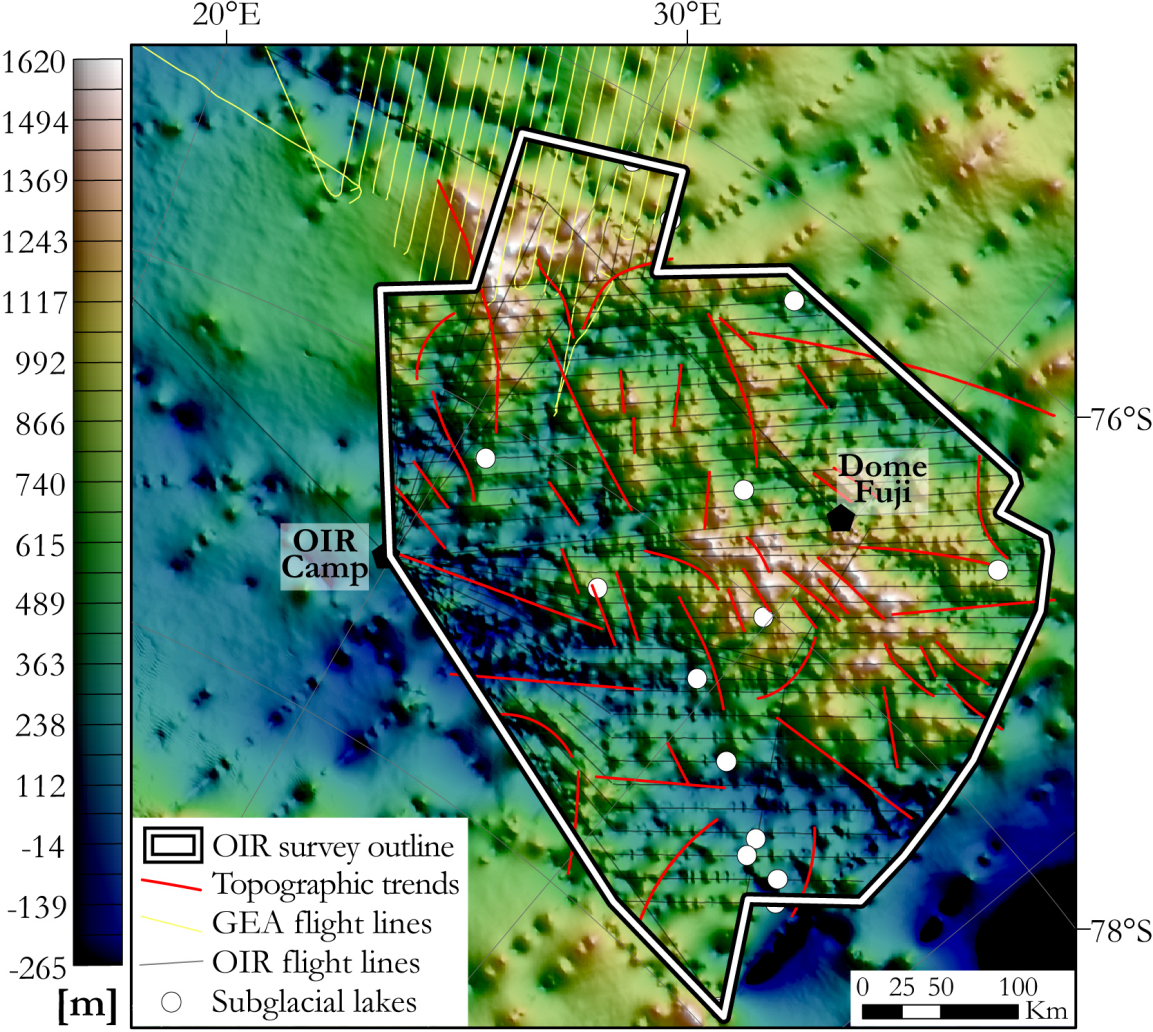
Subglacial topography and lakes calculated from the ice penetrating radar measured from the OIR and GEA-IV surveys^52^. Map shows the data as compiled within the BedMachine Antarctica data set^33^. Map was built using ArcMap 10.6.1.

Figure 3 shows the radar-derived^33,52^﻿ subglacial landscape of the OIR area to vary from 265 m below sea level to 1620 m above sea level (asl). Up to sixteen subglacial lakes were identified in the data^52^ and used to refine geothermal heat flux estimates^53^. The area is divisible into two topographic domains. North of 78°15’S lies a domain of prominent subglacial highlands reaching above 1600 m asl in its centre and northwestern parts, which is crossed by a network of V-shaped valleys as deep as 1000 m. The more subdued and generally lower (~ sea level) topography further south supports the recent inference of a subglacial sedimentary basin, the south Lambert basin, to the east^54^.

### Magnetic domains and lineaments

**Figure 4:**
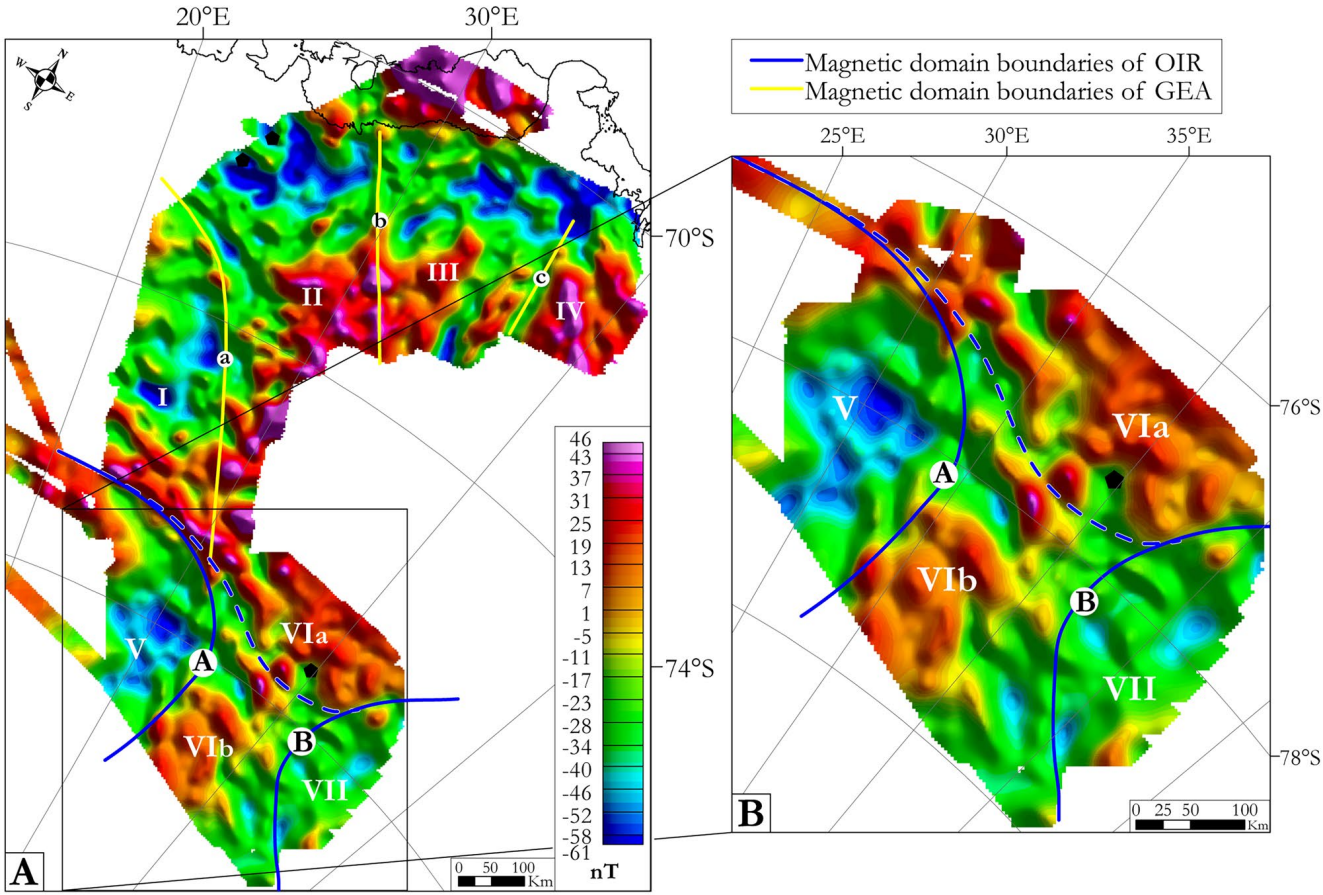
Magnetic data after upward continuation to 10 km to enhance signatures from deeper crustal sources. A: OIR and GEA survey regions. B: zoom in on OIR area. The dashed blue curve is the boundary between subdomains VIa and VIb. Maps were built using Geosoft Oasis Montaj 9.6.

OIR magnetic data were combined (see Methods) with contiguous earlier GEA datasets and interpreted within the existing GEA scheme^11^ (Fig. 4A). The highest anomaly values in the OIR survey area are situated in its central part, framed to the west by magnetic lows and to the east by intermediate strength anomalies. These trends are confirmed by upward continuation to 10 km, which enhances deep magnetic structures, allowing to expand the GEA interpretation scheme^11^ by the addition of three new domains, ‘V–VII’, separated by two curvilinear boundaries, A and B (Fig. 4B). ‘A’ is a sharp gradient that bends northwards from a N–S into an E–W orientation. ‘B’ shows opposing curvature, its orientation bends northwards from NW–SE to NE–SW. The western domain, ‘V’, is characterized by low amplitude, low frequency magnetic anomalies and is a continuation and enlargement of domain Ib^9^ in the GEA area. The central domain, ‘VI’, is identified by its magnetic highs and can be subdivided into two sub-domains based on comparisons with existing data. Sub-domain ‘VIa’, based on its long NW–SE-oriented magnetic highs, appears to be a continuation of GEA’s domain ‘II’. Sub-domain ‘VIb’ differs only slightly, its NW–SE-oriented magnetic highs being shorter. Domain ‘VII’ is characterized by low to intermediate magnetic signals.

**Figure 5:**
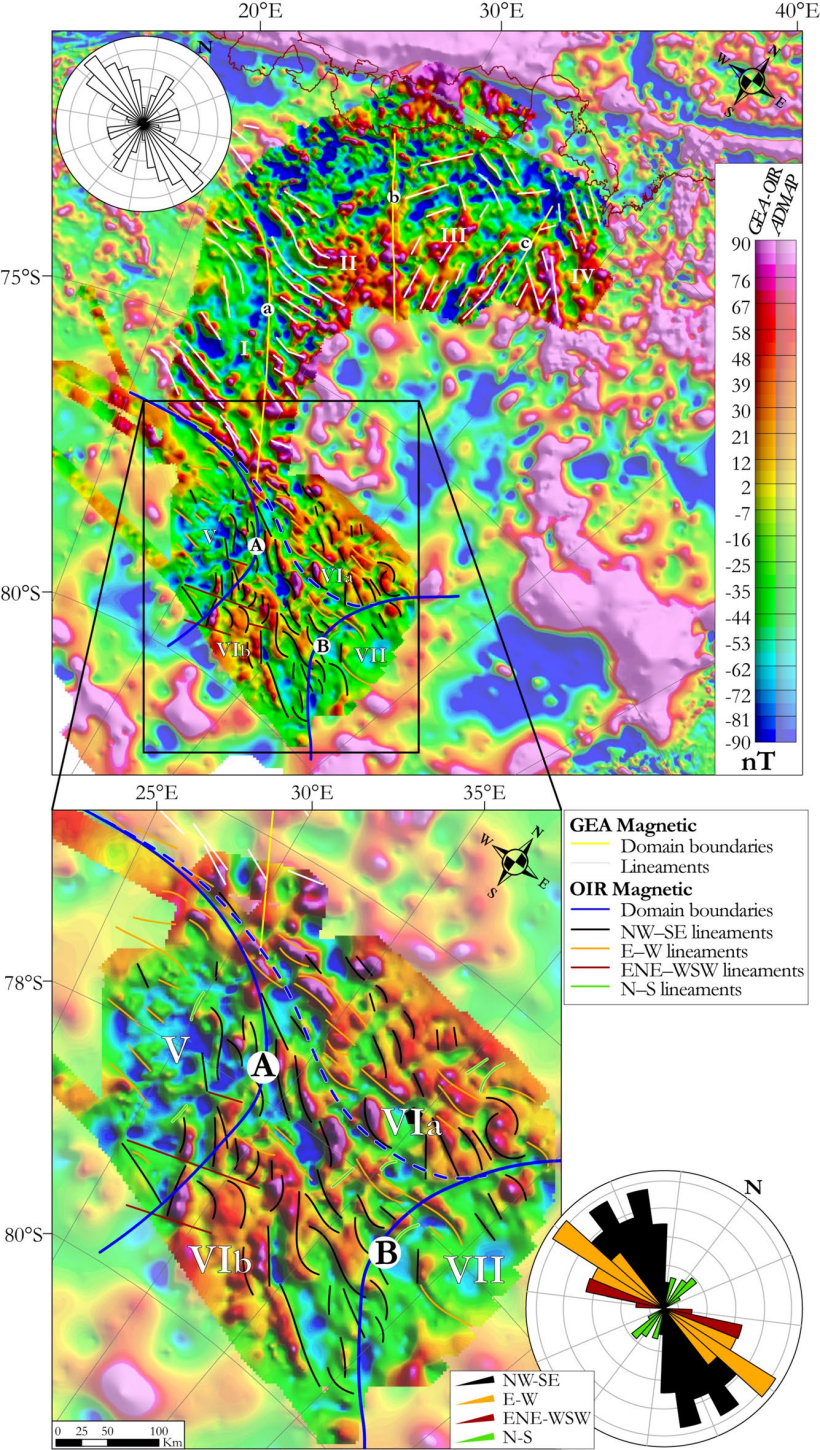
Total intensity magnetic anomaly map of OIR and GEA superimposed on the ADMAP magnetic map (paler colours). Results of GEA and OIR domain and lineament analyses are overlaid on the grids and summarized by rose diagrams. Maps were built using Geosoft Oasis Montaj 9.6.

Enhancements by tilt derivative and 3D Euler deconvolution techniques (see Methods for details) allow us to distinguish four dominant lineament trends in the combined OIR/GEA magnetic anomaly map (Fig. 5). These trends are overlaid on our OIR/GEA total intensity magnetic anomalies, and the surrounding continental-scale ADMAP-2 compilation, in Fig. 5 and described here: (1) NW–SE-oriented lineaments, mostly of moderate amplitude, 20–75 km long, and most common in domains ‘V’ and ‘VII’; (2) E–W-oriented lineaments typical of domains ‘V’ and ‘VIa’, of moderate amplitude or stronger in domain ‘VIa’, which lie close and subparallel to a sharp E–W-trending anomaly gradient^11^; (3) long (some exceeding 200 km) ENE–WSW-oriented lineaments, with peak amplitudes in the OIR data, and which define offsets in surrounding anomalies; (4) short, rare, N–S-trending lineaments of intermediate anomaly strength.

Overlaying our findings on the ADMAP-2 dataset^18^ (Fig. 5) shows how the NW–SE-oriented lineaments define a continuation of the magnetic “SE Dronning Maud Land province”^9^.The distinctive NW–SE-trending anomalies are relatable via outcrop to deformed granitoids in the TOAST. In the GEA and ADMAP-2 data, these anomalies are somewhat straighter than in the OIR area. These distinctive TOAST anomalies terminate in the southern GEA survey region at a sharp E–W-trending gradient^11^, which continues as boundary ‘A’ into the OIR region.

### Gravity domains and lineaments

**Figure 6:**
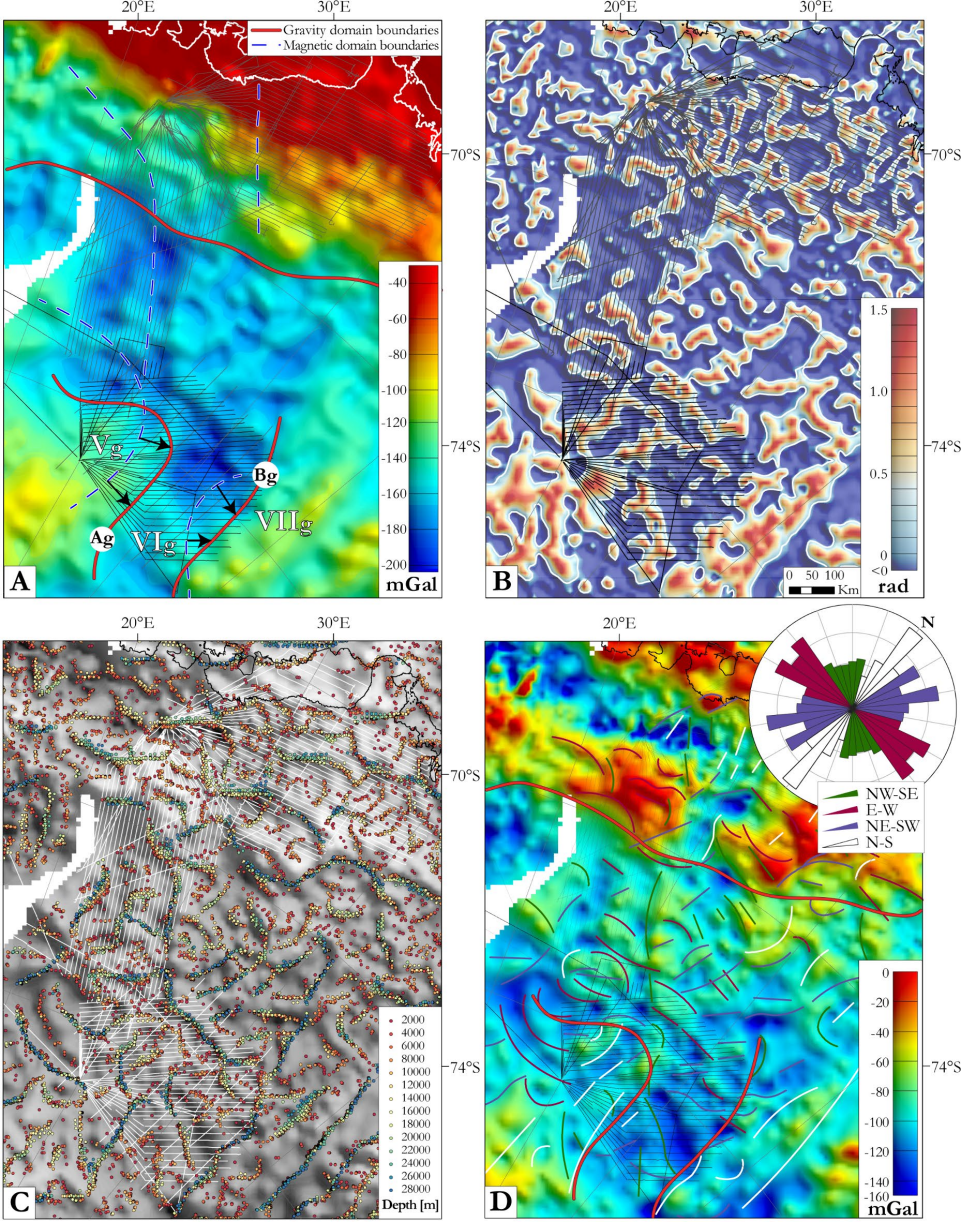
Gravity data filtering and enhancement. (**A**) Bouguer gravity anomalies after upward continuation to 10 km. (**B**) Positive tilt derivative of the Bouguer gravity anomalies to enhance lineaments and shallow sources with the zero contours in white. (**C**) Maxima of horizontal gradient of the Bouguer anomalies for different upward continuations overlaid on the isostatic residual gravity anomaly map in greyscale. (**D**) Resulting gravity lineaments superimposed on the isostatic residual gravity anomaly map. Rose diagram summarizes the lineaments. Maps were built using Geosoft Oasis Montaj 9.6 and ArcMap 10.6.1.

We complemented our magnetic interpretations using Bouguer anomalies sampled from the ANTGG data set^55^ (see Methods). In the OIR survey area, the gravity signal presents a quite homogeneous pattern of intermediate amplitude and frequency variation. Upward continuation to 10 km (Fig. 6A) helps to identify three gravity domains, from west to east ‘Vg’, ‘VIg’, and ‘VIIg’ in the OIR survey area. The three domains are elongated roughly N–S and delineated by boundaries ‘Ag’ and ‘Bg’. ‘Ag’ curves northwards from a N–S-orientation to E–W. ‘Bg’, near the eastern edge of the OIR area is N–S-oriented. Domain ‘VIg’, in the central/northwestern OIR survey area, is characterized by its Bouguer gravity low. Domains ‘Vg’ and ‘VIIg’ present intermediate strength signals. Two gravity domains over the GEA survey area are delineated by an E–W-oriented boundary. The northern domain is characterized by high amplitude, low frequency highs and the southern domain by low amplitude, low frequency lows.

Four groups of gravity lineaments are observable after enhancements via the tilt derivative and maxima of horizontal gradient carried out on different upward continuations (Fig.6B,C). Figure 6D summarizes the results, overlain on an isostatic residual anomaly map that serves to suppress long wavelength signals related to topography at the base of the crust: (1) ~ 25–100 km-long N–S lineaments, numerous in the northern GEA data towards the coast, and with different lengths (45–500 km) in the OIR domains ‘Vg’ and ‘VIIg’; (2) E–W-oriented lineaments averaging 150 km in length, continuous and evenly distributed over the GEA and OIR surveys but vanishing eastwards within them; (3) 30–160 km-long NE–SW-oriented lineaments that are homogeneously distributed over the GEA and OIR surveys; (4) Rare NW–SE-oriented lineaments, distributed homogeneously over the GEA survey area and domain ‘VIg’, but barely present in ‘Vg’ and VIIg’.

## Basement structures and correlations

### Correlations between subglacial topography and magnetic anomalies

Geological structures are often expressed in landscape morphology as linear ridges, terraces, or valleys. We identified topographic lineaments using the BedMachine Antarctica grid^33^ (Fig. 3), and compared the results to those of the magnetic lineament analysis (Fig. 5). In general, whilst individual magnetic and topographic lineaments tend not to correlate, a shared overall NW–SE trend is clearly evident. This suggests that subglacial topography in the OIR area is controlled by a widespread NW–SE-trending structural fabric. In addition, steep E–W-oriented slopes over the highest subglacial topography correlate with magnetic offsets, and so can be interpreted as expressions of E–W-oriented faults. Similar relationships between steep subglacial slopes and magnetic lineaments trending ENE–WSW are also interpretable in terms of a population of ENE–WSW striking faults (Fig. 5). A few long NE–SW-oriented topographic lineaments in the south and north of the OIR area do not correlate with any magnetic lineaments.

### Correlations between magnetic and gravity anomalies

Generally, it is accepted that, given the same resolution of acquisition, a region’s gravity signal gives information about crustal structures at greater depth than its magnetic signal. This expectation is even more likely to hold for our study because of the lower resolution of AntGG gravity data than the GEA-OIR magnetic data (see Methods). Consistent with this, it is difficult or impossible to routinely correlate individual gravity and magnetic signals between the data sets for the purposes of detailed geological interpretation. In the GEA survey area, however, some correlations between the magnetic or gravity signals and subglacial geology are possible at the domain scale given knowledge of the geology from outcrops at nunataks near the coast^11,56^. Hence, there is some scope to extend these correlations further into the GEA and OIR survey areas by tracing anomaly trends between them. To aid this task, we first removed crustal thickness signals from the gravity data by computing isostatic residual gravity anomalies (see Methods section).

The transition between the OIR and GEA survey areas shows a belt of positive magnetic anomalies that correlates with a belt of gravity highs (Figs. 5, 6). Within this pattern, a very good correlation can be observed between the NW–SE-trending positive magnetic anomaly corresponding to the contact between subdomains ‘Ia’ and ‘V’/‘Ib’ and a belt of intermediate gravity anomalies. Contrastingly, as already described, upward-continued gravity data over the GEA area reveal just two domains separated by an E–W boundary, but no correspondence to the three magnetic domains separated by NW–SE-oriented lineaments. Similarly, boundaries between the three magnetic domains are not evident via the gravity lineament analysis.

Further south, the two OIR magnetic domain boundaries seem to correlate with their gravity counterparts (Figs. 5, 6A), albeit shifted by ~ 80 km to the NNW. Assuming magnetic sources to be concentrated in the upper part of the crust, and gravity data to also reveal deeper components, the boundary offsets may indicate the presence of deep east-dipping crustal contacts between the domains. Within the OIR area, some good correlations can be observed between major gravity lineaments and successions of shorter magnetic ones. E–W-trending gravity lineaments correlate with E–W-oriented magnetic lineaments in the central (‘VI’/‘VIg’) domain of the OIR area where they indicate possible continuation of a trend that is also observed in the GEA area. Similarly, long NW–SE-oriented gravity lineaments within the central OIR domain correlate with successions of shorter NW–SE-oriented magnetic lineaments. The NE–SW-trending gravity lineaments, in contrast, have no magnetic counterparts. Finally, it is difficult to correlate the long N–S-oriented gravity lineaments within domains ‘Vg’ and ‘VIIg’ to their short N–S-trending magnetic lineaments.

## Discussion

**Figure 7:**
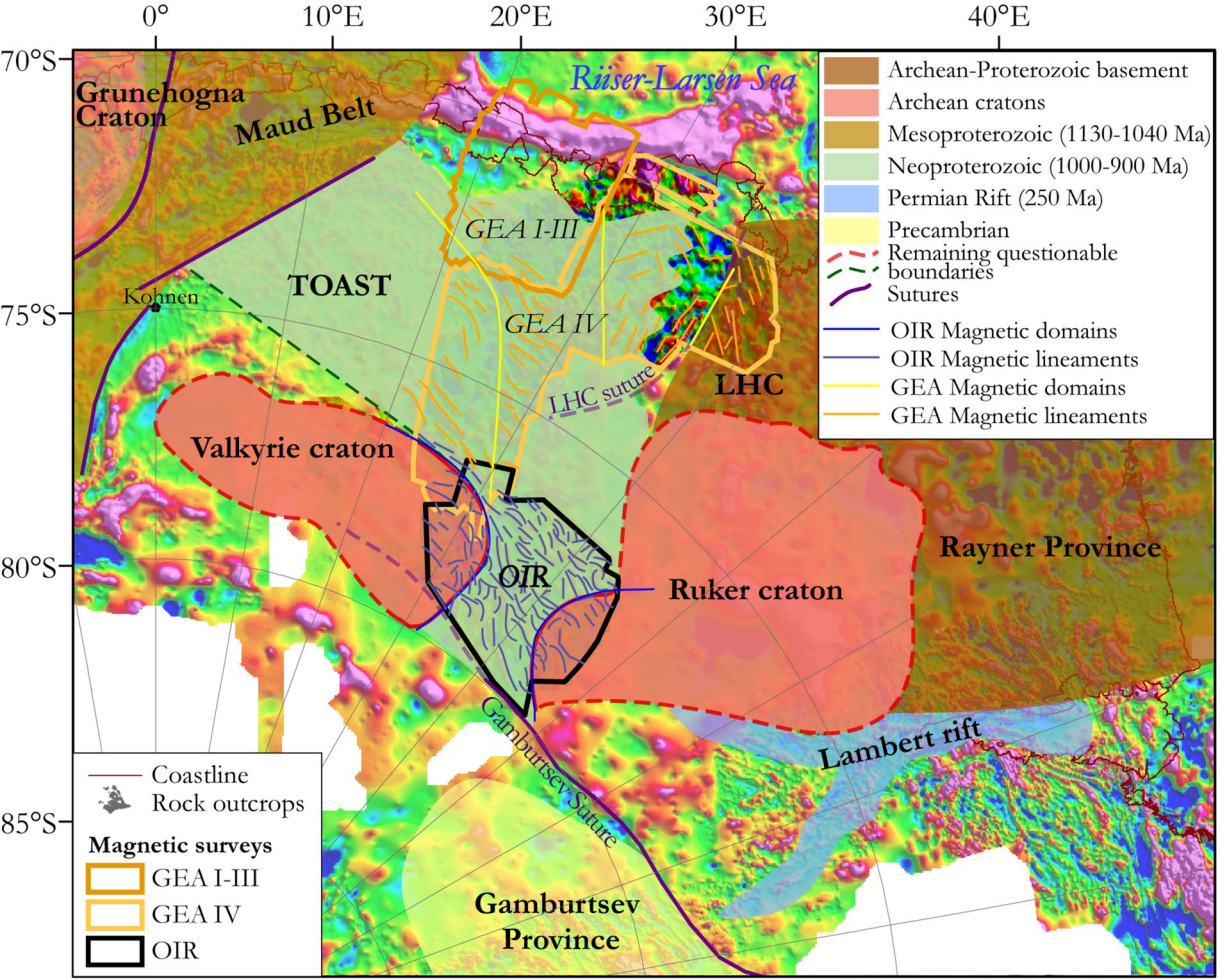
Revised tectonic interpretation map of Dronning Maud Land and western Enderby Land. Base image: ADMAP-2 compilation^18^. Map was built using ArcMap 10.6.1.

Our analyses of new and existing magnetic, subglacial topography, and gravity data and their enhancements and correlations support a set of preliminary new interpretations of subglacial tectonic structures in easternmost DML (Fig. 7). Based on these interpretations, the following sections examine the spatial extent of TOAST, the boundaries of the Valkyrie and Ruker cratons, and some newly identified tectonic structures between them. Finally, we contextualize our new interpretation by reference to existing continental-lithospheric scale studies.

### Southern extent of the Tonian Oceanic Arc Super Terrane

The TOAST, volcanic arc remnants of the Mozambique Ocean^24^ is typified at outcrop by the gabbro-tonalite-trondhjemite-granodiorite suites of the Sør Rondane Mts.^24,25^. In geophysical data, these remnants manifest as an association of semi-circular magnetic and gravity highs, which are interpreted as mafic intrusions^11^, and as a set of distinctive NW–SE-trending magnetic lineaments whose precise source configurations remain to be determined^9,11,18^. The widespread extent of this association over the ice-covered parts of central and southern DML reveals widespread Neoproterozoic crustal addition in the Mozambique Ocean prior to its subduction and the ensuing collision that generated the EAAO^11,18^.

In the OIR magnetic data, domain ‘VI’ is defined on the basis of its NW–SE-trending lineaments (Fig. 5). These lineaments are particularly well developed in subdomain ‘VIb’, whilst ‘VIa’ additionally shows numerous E–W-oriented magnetic lineaments that can be considered to continue the previously identified positive magnetic anomaly belt dividing subdomains ‘Ia’ and ‘Ib’ in the GEA area^11^. The NW–SE-trending magnetic patterns in domain ‘VI’ generally correlate with the global gravity lineament pattern of the domain ‘VIg’ (Figs. 5, 6). As the trend directions of both magnetic and gravity anomalies correspond to geophysical fabrics that elsewhere define the TOAST, we suggest that domains ‘VI’ and ‘VIg’ represent a southern continuation of the TOAST into the OIR survey area (Fig. 7). The area of this addition is approximately 120000 km^2^, or around 20–25% of the previously estimated area of the TOAST^9^.

### Boundaries of the Valkyrie and Ruker cratons

Domains ‘V’ and ‘VII’ display long wavelength negative magnetic anomalies associated with intermediate gravity signals (Figs. 4B, 6A), typical of cratons, that clearly contrast with the TOAST-like characteristics of domains ‘VI’ and ‘Ia’ in the OIR and GEA areas. Along the zone of contrast, the boundary between domains ‘Ia’ and ‘V’ is a belt of E–W positive magnetic anomalies (Fig. 5), previously identified as the contact of subdomains ‘Ia’ and ‘Ib’ in the GEA area^11^, which we name here the Elbert magnetic anomaly. This major (> 300 km long, ~ 30 km-wide) anomaly shows good correlation with an intermediate-amplitude gravity anomaly (Fig. 6D), forming a major geophysical feature that marks a suture between the Valkyrie craton^18^ and the TOAST. Although less pronounced, we have described how the eastern boundary of domain ‘V’ can be well delimited by an eastward increase in the amplitudes and frequencies of both magnetic and gravity anomalies (Figs. 5, 6), in which direction they become more TOAST-like. Based on these contrasts, we suggest that the margin of the Valkyrie craton follows the Elbert magnetic anomaly before bending into a N–S orientation within the OIR survey area (Fig. 7).

Domains ‘VI’ and ‘VII’ and their boundary can be interpreted in similar terms. The low amplitude and low frequency negative to intermediate magnetic anomalies of Domain VII are associated with intermediate amplitude and frequency gravity signals, all of which differ from the TOAST-like signals of domain ‘VI’ to the west. Away to the east of domain VII, the Ruker Craton is defined from outcropping basement rocks in the southern Lambert Rift^23,28,34^. Potentially, therefore, the magnetic and gravity boundary ‘B/Bg’ may represent part of the western boundary of the Ruker craton, where it meets the TOAST (Fig. 7).

The OIR data do not reveal features that might be interpretable in terms of the Gamburtsev Suture, the proposed southern boundary of the Ruker Craton to the northern Gamburtsev Province^34^. If the suture exists, therefore, it is likely to run south of the OIR survey area. Similarly, there is no specific signature in the OIR or GEA-IV datasets that might confirm the existence or location of the proposed Lützow-Holm Complex suture, a feature that was suggested on the basis of outcrop studies to mark the final amalgamation of Gondwana from eastern and western components^3,32^. If this second suture exists, therefore, it must be confined to the area east of the GEA-IV and north of the OIR survey areas, and so is likely to be of local rather than continent-wide significance.

### OIR Shear Zone: a TOAST-hosted collision between the Valkyrie and Ruker cratons?

**Figure 8:**
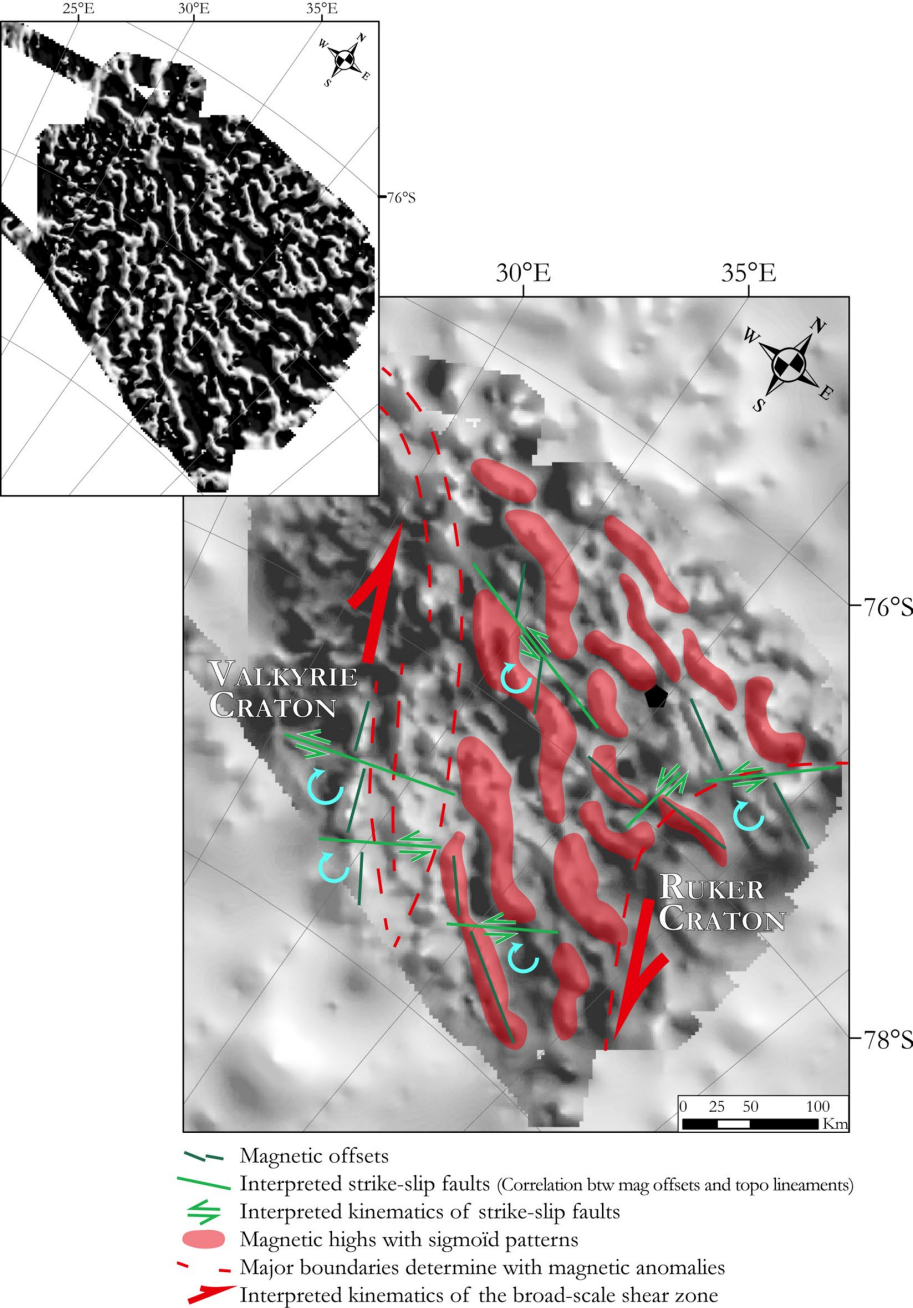
The OIR Shear Zone and its interpreted kinematics between the Valkyrie and Ruker cratons superimposed on the total field magnetic data (OIR survey and ADMAP-2). Blue curved arrows indicate the sense of rotation of individual fault-bounded blocks within the shear zone. Inset: tilt angle enhancement of the sigmoid structures. Maps were built using Geosoft Oasis Montaj 9.6 and ArcMap 10.6.1.

The magnetic anomaly pattern of domain ‘VIa’ differs slightly from that of domain ‘VIb’, suggesting some process has modified its TOAST-like structures. The tilt angle enhancement of magnetic data in Fig. 8 (inset) suggests how the modified structures can be described as a ~ 200 km-wide swath of sigmoidal magnetic sources, each defined by E–W-trending bars and NW–SE-trending arms. Assuming that the sigmoidal anomalies express sources that were originally linear and NW-trending, like those in the main body of the TOAST further north, their present shapes can be interpreted by analogy to an S-C fabric at outcrop scale (Fig. 8) as has been done previously for the Trans-Saharan belt between the West African Craton and the Saharan Craton^57,58^ or in the younger deformation zone between the North America, Pacific, and Bering plates^59^. This approach defines a history of dextral shearing affecting a narrow segment of the TOAST between the Valkyrie and Ruker cratons. We refer to this feature as the OIR Shear Zone.

Offsets of magnetic anomalies that mark the Valkyrie and Ruker craton margins correlate with sharp subglacial valleys that cross from the cratons into the OIR Shear Zone. The senses of these offsets suggest that the valleys should be interpreted as traces of sinistral strike-slip faults whose action would be consistent with the expected clockwise sense of rotation between individual fault-bounded blocks within a broader dextral OIR Shear Zone. Hence, although most of the OIR Shear Zone is accommodated within the TOAST, it seems that the cratons were present at its margins whilst it was active. The OIR Shear Zone thus appears to mark oblique collision of the Ruker and Valkyrie cratons (Fig. 8) during closure of the TOAST’s parental Mozambique Ocean^21,30^. The collision must therefore post-date early Neoproterozoic times. Consistent with this, dextral shearing has previously been interpreted to explain apparent deformation of TOAST anomalies further north in the GEA area^11^ where, on the basis of correlations to outcrop studies in the Sør Rondane Mts, it is also attributed to Pan-African events. Finally, given the OIR Shear Zone’s broader setting, it is possible to suggest an African affinity and west Gondwanan origin for the Valkyrie Craton.

### Comparison with previous large scale tectonic studies

In this section, we assess the wider setting and significance of our OIR Shear Zone with reference to four continental scale data sets and analyses drawn from the literature.

**Figure 9:**
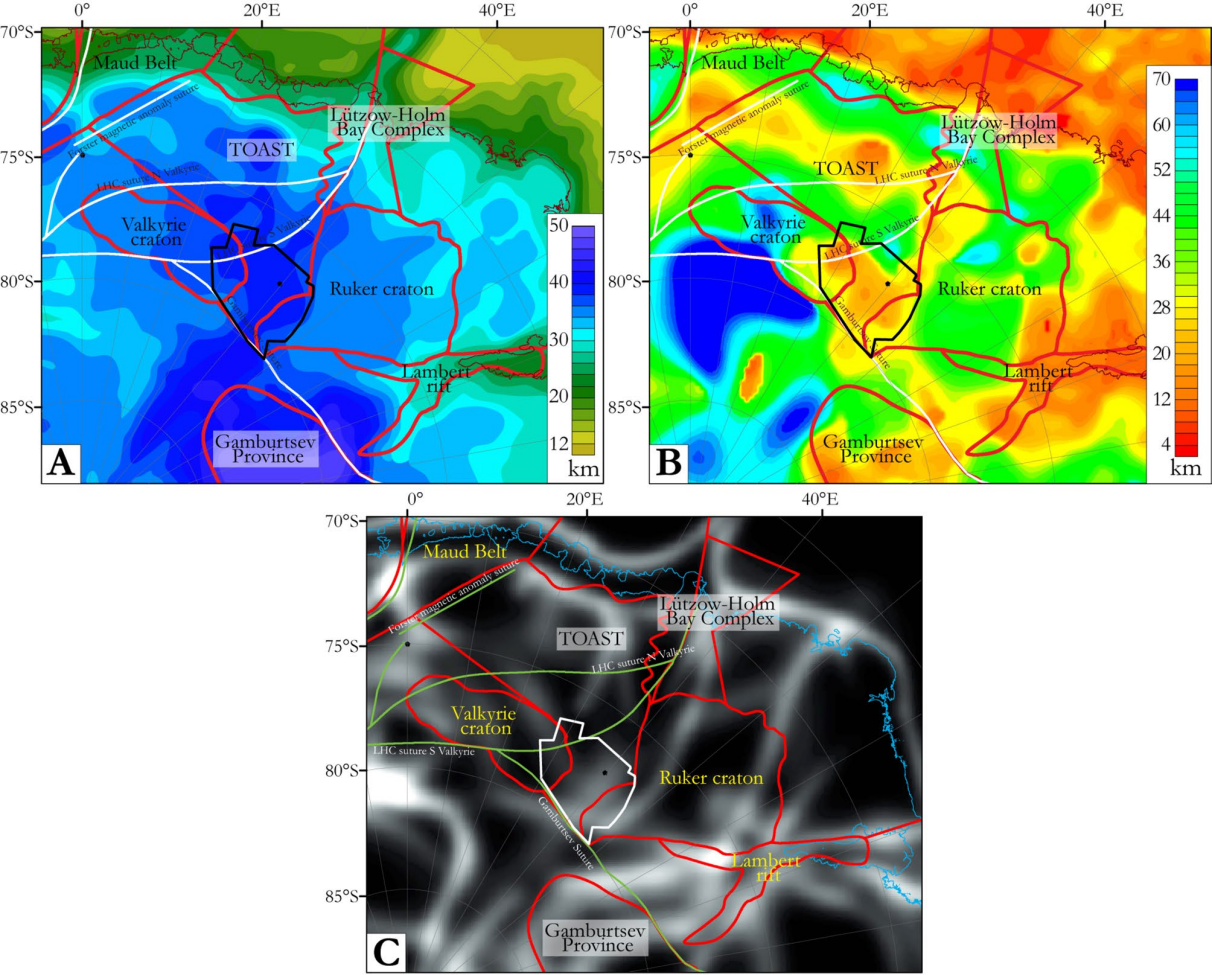
Comparisons of our interpretation with results from previous lithospheric studies. (**A**) Crustal thickness^61^. (**B**) Effective elastic thickness^62^. (**C**) Likelihood of tectonic boundaries via multi-variable statistical correlation^63^. New artwork created for this manuscript using Geosoft Oasis Montaj 9.6 and ArcMap 10.6.1.

First, we note that the N–S trend of the OIR Shear Zone is mirrored in sparse mantle seismic anisotropy orientations, which form along a southwards continuation of its strike^60^. Relative motion on the shear zone thus likely affected the entire lithosphere. Second, the crustal thickness of East Antarctica, based on seismological and petrological models combined with GOCE satellite gravity gradient data, ranges from 38 to 42 km over the OIR survey area, but is slightly thinner, at ~ 35 km^61^ to the east and west. The OIR Shear Zone thus occupies a narrow N–S-trending strip of thicker crust, which Fig. 9A shows to connect the TOAST southwards to the Gamburtsev Province, and may continue further to the Transantarctic Mountains^61^. Third, an effective elastic thickness (Te) map obtained by the Bouguer coherence technique^62^ shows a similar corridor, this time in the form of low Te values in the TOAST sandwiched between the thicker neighbouring Valkyrie and the Ruker cratons (Fig. 9B). Fourth, a feature parallel to the OIR Shear Zone, but shifted slightly to the east of it, also appears in likelihood maps (Fig. 9C) generated via a statistical correlation approach to synthesizing seismic, gravity and surface elevation data and validated by surface expressions of crustal tectonic boundaries exposed along the coast^63^.

Based on all of these observations, it is reasonable to interpret the OIR Shear Zone as the remnant of a late Neoproterozoic plate boundary segment that ran through the TOAST between the Valkyrie and Ruker cratons and, further south, somewhat more speculatively between the Transantarctic Mountains and Gamburtsev Province. Whilst other interpretations of its role might come to be considered in the light of new data, it seems most likely for now that this plate boundary segment was active during the closure of the Mozambique Ocean.

## Conclusions

Combined analyses of new and existing subglacial topography, gravity and magnetic data in the region of Dome Fuji in eastern DML help to refine the tectonic map of East Antarctica. Topographic, gravity and magnetic lineaments were identified and correlated at a regional scale to reveal prominent regional NW–SE-trends that suggest a southwards continuation of the TOAST beyond its previous known extent. In addition, a prominent E–W-oriented 300 km long belt of positive magnetic anomalies is identified and named the Elbert magnetic anomaly, and interpreted to mark a Pan-African suture between the subglacial Valkyrie craton and the TOAST. The eastern and western margins of the Valkyrie and Ruker cratons are identified as possibly-east-dipping contacts bounding a narrow (~ 200 km wide) neck of deformed TOAST material. In addition, sigmoid shapes and offsets of some positive magnetic anomalies within the neck are associated with the gravity- and seismic- evidence for thick crust, leading us to the interpretation of a broad scale shear zone between the two cratons, the OIR Shear Zone, a new feature of significance for studies of Gondwana’s amalgamation.

## Methods

### New magnetic data

**Figure 10:**
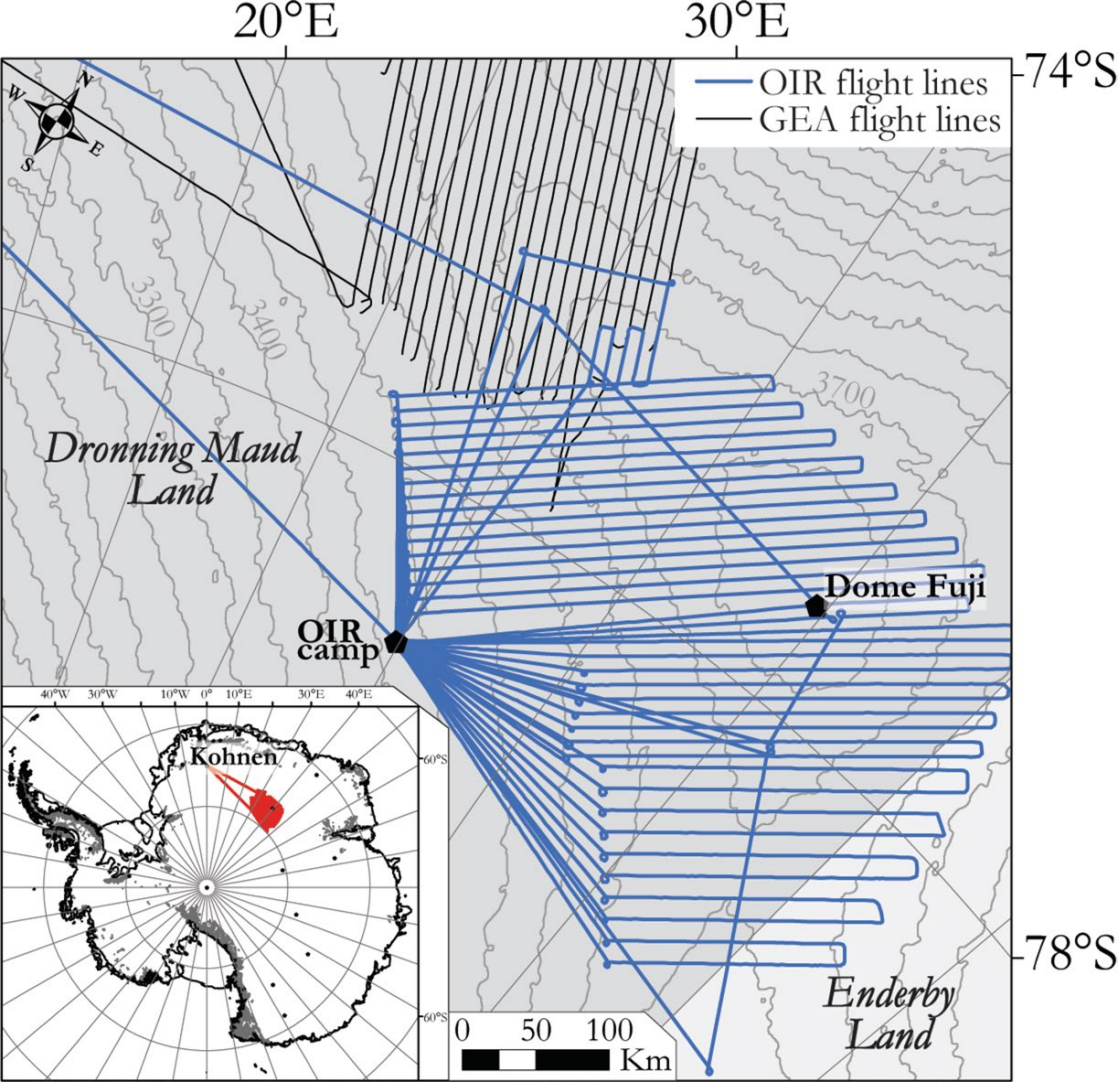
OIR survey flight lines (red in the inset, blue otherwise) and GEA survey flight lines. Grey curved lines are the isocontours of the topography of the ice surface. In inset is the location of the study area at the scale of Antarctica. Map generated using ArcMap 10.6.1.

The OIR aerogeophysical survey was performed during the Alfred Wegener Institute’s Antarctic summer campaign of 2016–2017 in the vicinity of Dome Fuji in East Antarctica (Fig. 10) as part of the European project “Beyond EPICA”. The primary goal of the OIR campaign was the characterization of a possible site for drilling Antarctica’s oldest ice in order to generate a continuous 1 to 2 million year-long continuous climate record^52^. Two radar systems measured the thickness of the ice sheet, supplemented by video and optical cameras for recording the surface of the ice. A magnetometer was also carried to get an insight into the geological characteristics of this fully ice-covered region. The only previous airborne magnetic survey of the region was completed by the Soviet Antarctic expeditions in the 1960’s and 70’s in the Dome Fuji region^64,65^. Those data were acquired at higher altitude (2500 m above the ice surface), on more widely-spaced tracks (ca. 50 km^27^), and could not benefit from precise global satellite navigation systems, thus having large positioning errors. These older data are therefore not very suitable for detailed geological interpretation.

The OIR survey was completed as part of the austral summer season during December 2016 and January 2017. The survey was located between 76°S/25°E and 80°S/48°E, covering an area of ~ 170000 km^2^ with ~ 23 000 line-km of new data (Fig. 10). In total, data were acquired during 24 flights around Dome Fuji. Most of the lines were spaced at 10 km, except for the five southern flights where the spacing was 15 km. The flights were carried out with an average ground speed of 260 km/h and at 450 m height above the ice sheet. The orientation of the parallel flight lines was NE–SW. Four tie lines were flown to enable cross point analysis and levelling. Data were recorded at 1 s intervals resulting in an approximate along-line point spacing of 75 m. The flight paths were post-processed using differential GPS procedures. The aircraft was equipped with a Scintrex Cs-3 caesium vapour magnetometer mounted in a tail boom and two radar systems^52^. In addition, a three-component Billingsley TFM100 fluxgate magnetometer was mounted in the fuselage to enable the magnetic compensation. In the absence of a nearby magnetic observatory, a magnetic base station was established at the temporary base camp to record the diurnal variation in the vicinity of the survey area for removal from the flight records.

**Figure 11:**
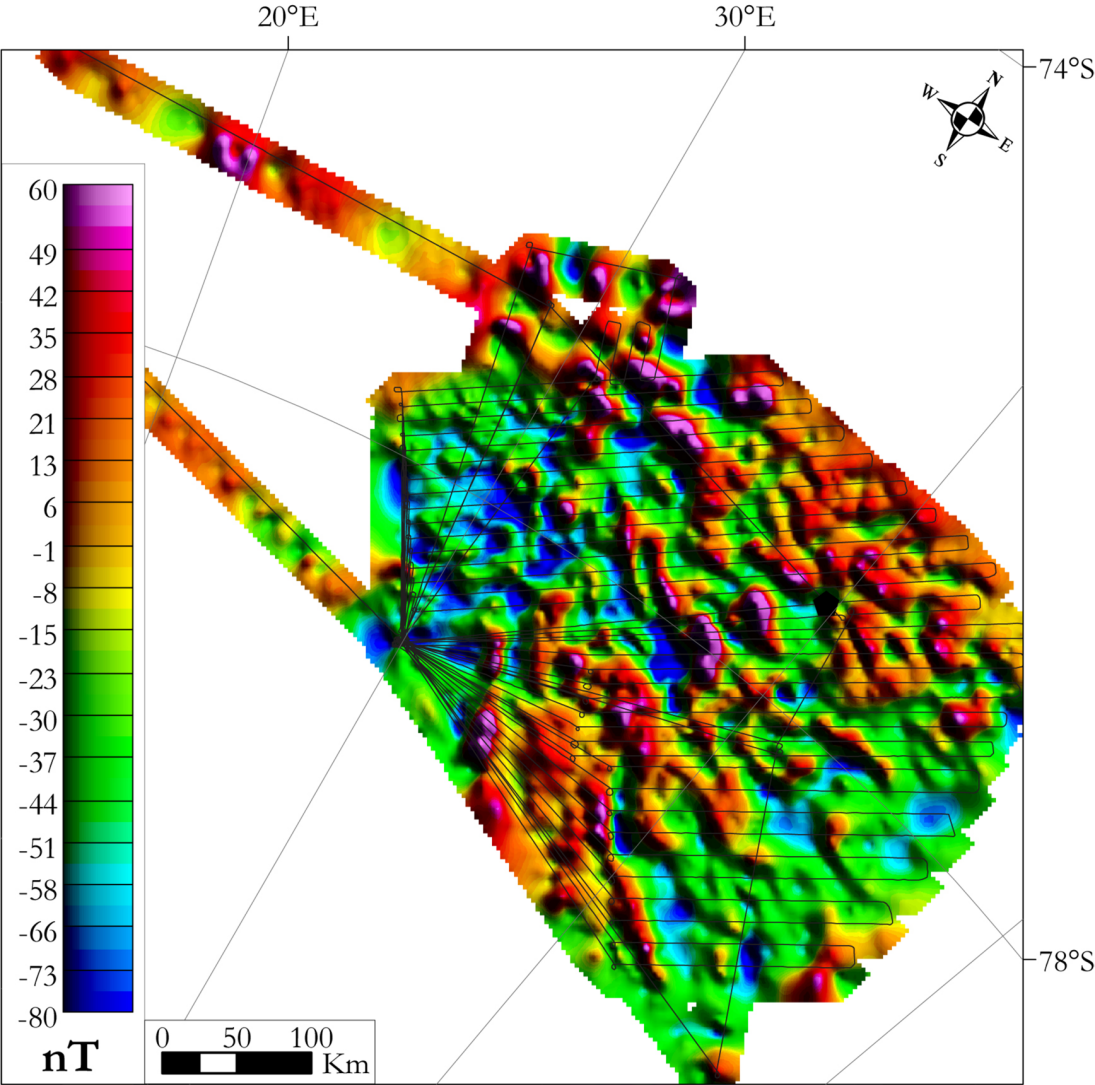
Total magnetic intensity map of the OIR survey reduced to the pole (linear scale) with survey flight lines superimposed. Map generated using Geosoft Oasis Montaj 9.6.

The airborne magnetic data were de-spiked, IGRF-corrected, and corrected for diurnal variations recorded at the base station, by using Geosoft’s *Oasis montaj* software. Diurnal variations were recorded in intervals of 5 s, then quality checked and low-pass filtered at a cutoff of 1200 s to remove the high frequency signal due to the high magnetic activity level in Antarctica. Owing to the relatively sparse distribution of tie lines and the presence of strong aircraft and heading effects in the data, following a malfunction of the fluxgate magnetometer, the levelling strategy was to replace the long-wavelength component of the recorded data with the corresponding component from another source. We chose the Magnetic Field model MF7^66^ for this purpose, because it provides a good balance between data resolution, noise and coverage. The choice unfortunately, but unavoidably, leads to an product that completely suppresses wavelengths in the 180–300 km range^67^. Finally, the corrected and levelled magnetic data were gridded using the minimum curvature method with a grid cell size of 3 km and reduced to the pole using a mean declination of -50.8° and a mean inclination of −66.5° (Fig. 11).

### Previously acquired and previously published datasets

We support and enhance our analysis of OIR magnetic data using existing additional datasets for bed topography, gravity, and adjacent magnetic surveys. These complementary data are summarized in Table 1.

**Table 1:** Summary of datasets used in this study including previously acquired data.

Name	Data type	Data content	Grid resolution	Reference
BedMachine	Subglacial topograpy	Compilation of variety of sources	500 × 500 m	Morlighem et al.^33^
OIR radar	Subglacial topography	Airborne radar survey	1 × 1 km	Karlsson et al.^52^
ADMAP2	Magnetic	Compilation of different airborne magnetic surveys	1,5 × 1,5 km	Golynsky et al.^18^
GEA I-III-IV	Magnetic	Airborne magnetic surveys	3 × 3 km	Ruppel et al.^11^
AntGG	Gravity	Compilation of different terrestrial, airborne, and shipborne gravity surveys	10 × 10 km	Scheinert et al.^55^

#### Subglacial topography

Subglacial topography provides insights into the landscape of the area by revealing the presence of buried mountain ranges, valleys, basins, crests and troughs. In areas without surface outcrop, the correlation between topography and magnetic anomalies allows an assessment of the possible locations of major faults, via the detection of scarps and valleys that form along lithological contrasts created by shear zones. In addition, a digital elevation model is essential for correcting gravity data for the effects of topography. We used an extract of the BedMachine Antarctica subglacial topography^33^ for the Dome Fuji region (Fig. 3). The BedMachine grid is based on a compilation of a variety of sources such as satellite altimetry, direct ice thickness measurements, and synthetic ice thicknesses, presented at a spatial resolution of 500 m × 500 m. The grid is locally constrained and enhanced by the recognition that rates of ice motion, constrained by near-surface observations and physical models of ice sheet flow, must be modulated by the shape of the underlying rock surface. The BedMachine dataset in the Dome Fuji region includes the OIR and GEA radar data, which were previously published elsewhere^52^. We analysed these data for lineaments by illuminating them from various declinations with a fixed inclination of 45°, which visually enhances steep slopes, discontinuities and offsets by variations of shadow on the topography.

#### Existing magnetic data

**Figure 12:**
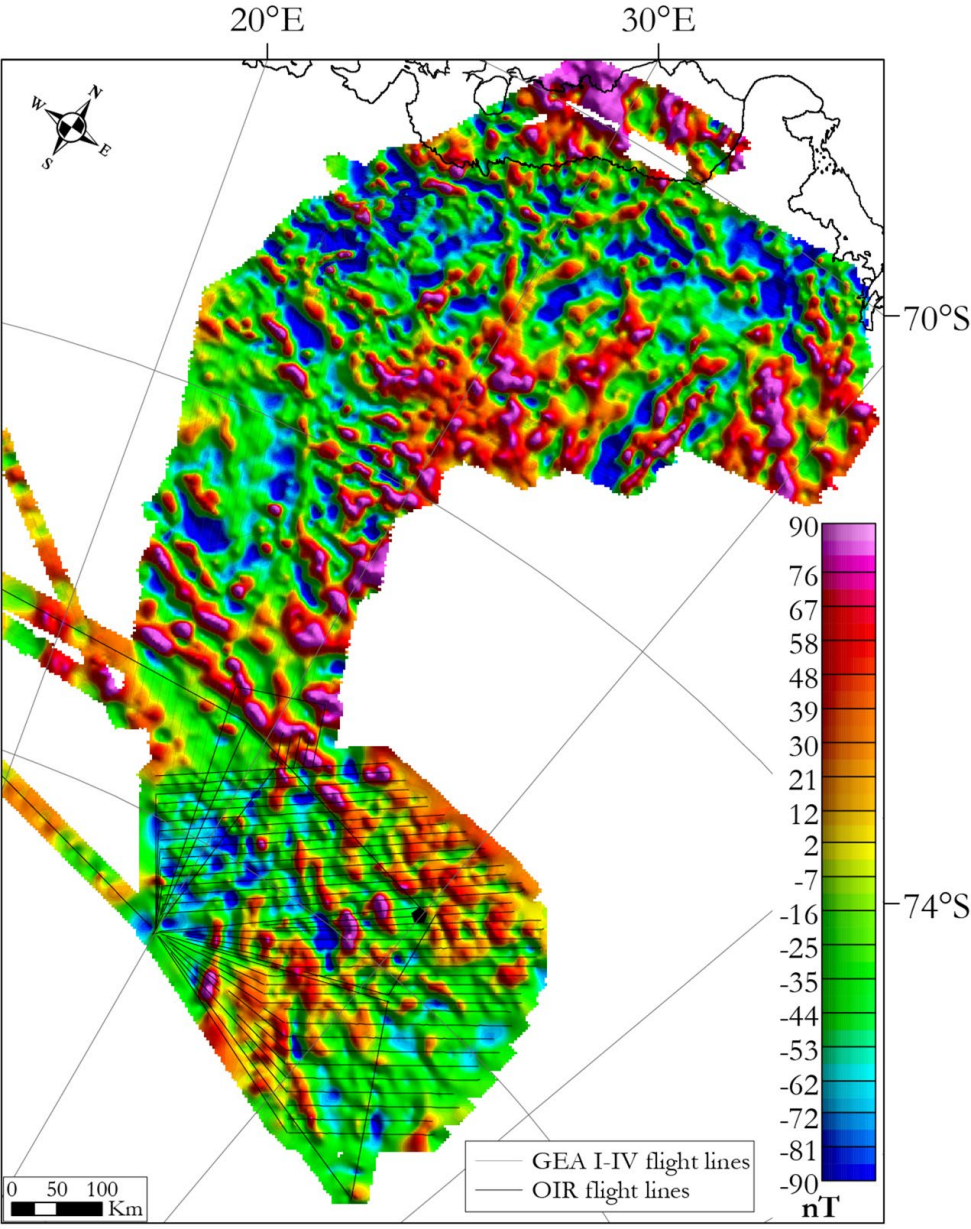
Total magnetic intensity map, reduced to the pole, showing merged OIR and GEA I–IV surveys (linear scale) with survey flight lines superimposed. Map generated using Geosoft Oasis Montaj 9.6.

The new OIR magnetic data were compiled together with the neighbouring GEA and wider ADMAP-2 datasets in order to investigate the possible continuations of major magnetic domains and magnetic lineaments in the broader region. The ADMAP-2 dataset comprises 3.5 million line-km of airborne and shipborne data, gridded at a consistent spatial resolution of 1.5 km^18^ in spite of very wide survey line spacing in the Dome Fuji region. ADMAP-2 contains all available existing data in the areas to the east-, west- and south of the OIR survey area from the period until 2015. The region to the north of the study area was more recently surveyed as part of the collaborative Geodynamic Evolution of East Antarctica (GEA) surveys by the German Federal Institute for Geosciences and Resources and the Alfred Wegener Institute. These surveys cover the area to the south and east of Sør Rondane Mountains consisting in 40 000 line-km of data acquired in 2011/12, 2013/14 and 2014/15 austral summers, gridded and merged at a spatial resolution of 3 km^11,68^, most of which were not included in the ADMAP-2 compilation. We levelled, gridded and merged the GEA data together with the OIR magnetic to produce a more extensive total intensity magnetic anomaly map at a spatial resolution of 3 km (Fig. 12). The same data filtering procedures were applied to this magnetic map as for the OIR magnetic map.

#### Gravity data

**Figure 13:**
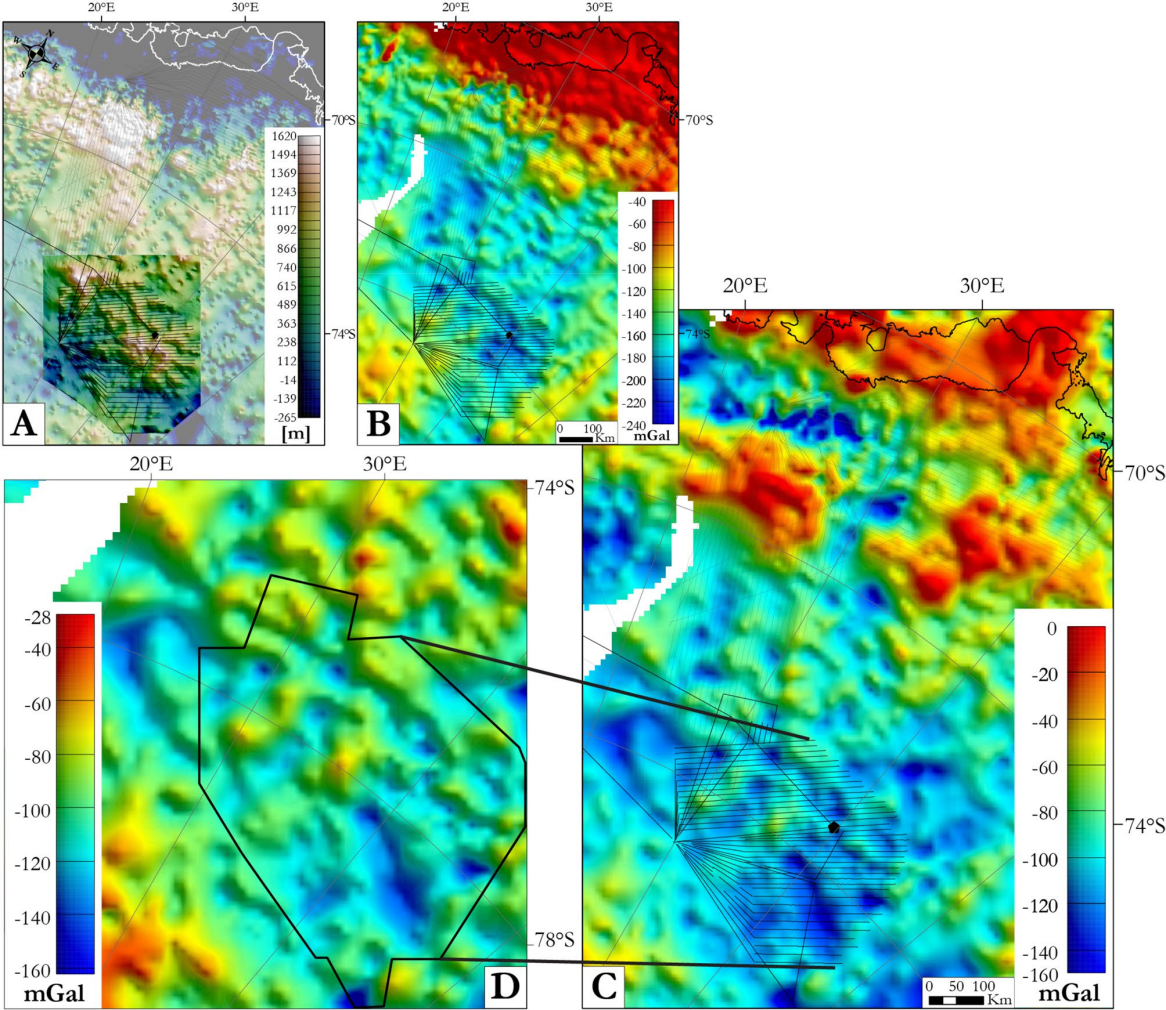
Computation of the isostatic residual anomalies from the AntGG and BedMachine grids. (**A**) OIR bed rock topography merge with the BedMachine topographic model^33^. (**B**) Bouguer anomaly map extracted from the model ANTGG^55^ over the GEA and OIR magnetic survey areas. (**C**) Isostatic residual anomaly map. (**D**) Zoom on the isostatic gravity residual map over the OIR survey area. Maps generated using Geosoft Oasis Montaj 9.6.

The analysis of magnetic anomalies in combination with the gravity signal is essential to better constrain the structure of the crust and decipher the distributions of terranes and continental blocks. No new gravity data were collected during the OIR survey. Instead, we used the “Gravity and Geoid in Antarctica” (AntGG) grid, which is a compilation of ground-based, airborne and shipborne gravity data derived from 13 million data points acquired before 2015 and covering 73% of the Antarctic continent^55^. The grid was computed using terrain and topographic corrections generated using the Bedmap2 subglacial topography (Fig. 13A) and ice thickness compilations^69^ to obtain complete Bouguer anomalies at a spatial resolution of 10 km. Newer, higher resolution gravity data do exist for the GEA-IV area^6^. Their long-wavelength components are similar to the lower resolution AntGG grid. After making this observation, we did not use the GEA-IV gravity data further here because of the absence of similar high-resolution data over the OIR survey area. The extracted complete Bouguer anomaly data over the OIR and GEA survey areas are shown in (Fig. 13B). The subglacial topography map (Figs. 3, 13A) enabled the computation of isostatic residual gravity anomalies over the GEA and OIR surveys using the Airy-Heiskanen compensation model with a compensation depth at 32 km for topography at sea level, a Moho density contrast of 330 kg/m^3^, and crustal density of 2670 kg/m^3^ (Fig. 13C,D). This procedure removes the long-wavelength gravity anomalies, which are related to deep sources such as the variation of the Moho boundary. Thus, signals from intracrustal gravity sources are enhanced, enabling more confident comparison with the magnetic signal.

### Data enhancements and transformations

**Figure 14:**
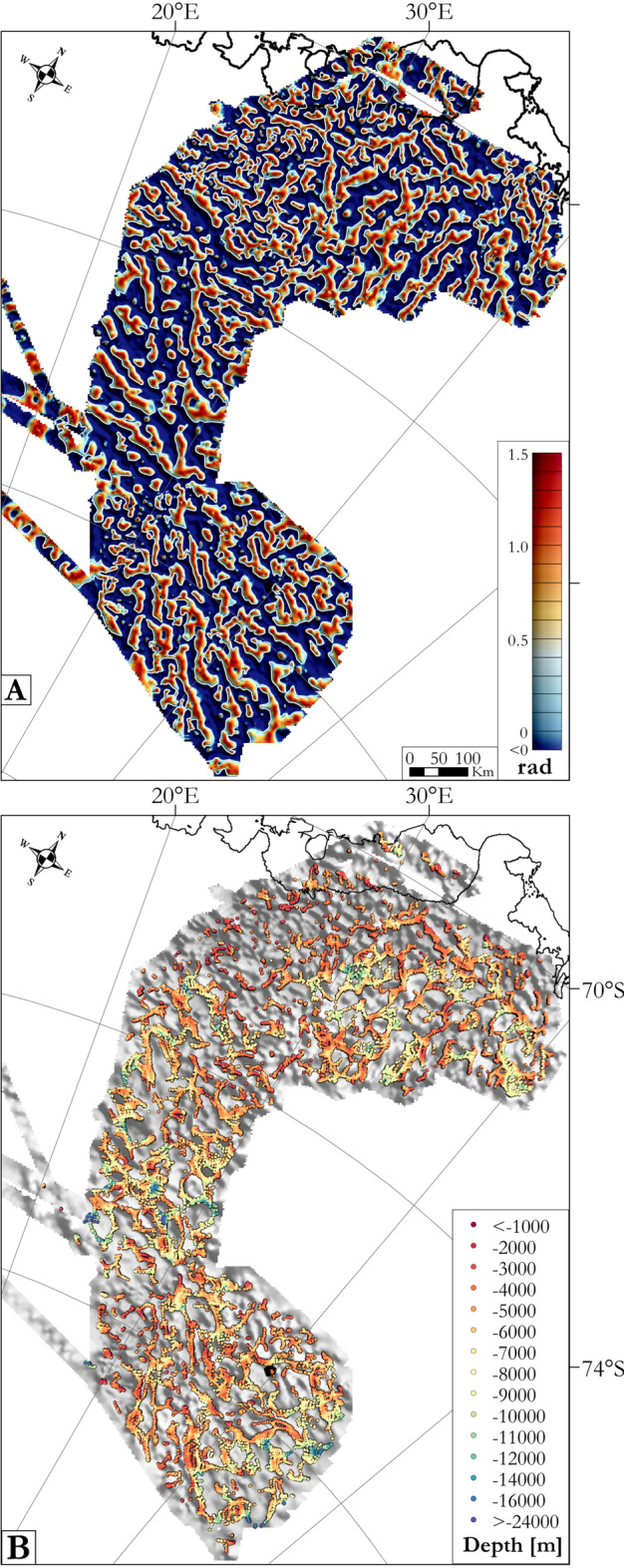
Results of magnetic data filtering to enhance signals from shallow sources and estimate depths to sources. (**A**) Positive tilt derivative of the magnetic anomalies to enhance lineaments and shallow sources with the zero contours in white. (**B**) Results of 3D Euler deconvolution indicating the depths of magnetic source bodies superimposed on magnetic map in grey. Maps generated using Geosoft Oasis Montaj 9.6.

Enhanced data filtering procedures and transformation techniques were applied to determine the trends and locations of the magnetic lineaments, the source depths and their possible geometries (Fig. 14). An upward continuation to 10 km above sea level was performed to enhance signatures from deeper crustal sources and to highlight magnetic domains^70,71^. Reduction to the pole transforms magnetic anomalies, under the assumption they are dominated by induced components, so that they become symmetrical and align with their sources in the sub-surface. The positive tilt derivative of these transformed magnetic anomalies enhances the trends of lineaments and anomalies due to shallow sources^72,73^ (Fig. 14A), and its zero contour approximates the edges and shapes of causative magnetic sources^74^, which thus map the structural patterns of the upper crust. Finally, 3D standard Euler deconvolution was performed^75^ (Fig. 14B). In the absence of outcrops and seismic data over the OIR area, we used a large window size of 30 km and default structural index of 1. The aim of this step was to identify trends in the orientation and distribution of the magnetic fabrics, rather than their depths. We compared our results with those obtained by a more detailed Located Euler Depth analysis for the GEA area^11^. We noticed shared trends in the two sets of results, and also that our solutions were more numerous, as might be expected of the less restrictive input parameters used.

## Data Availability

All published data are available via the cited references. The GEA and OIR magnetic line data will become available in a forthcoming update to the ADMAP compilation.
